# The Effect of Balsamic Vinegar Dressing on Protein and Carbohydrate Digestibility is Dependent on the Food Matrix

**DOI:** 10.3390/foods10020411

**Published:** 2021-02-12

**Authors:** Eleonora Urbinati, Mattia Di Nunzio, Gianfranco Picone, Elena Chiarello, Alessandra Bordoni, Francesco Capozzi

**Affiliations:** 1 Department of Agri-Food Sciences and Technologies (DISTAL), University of Bologna, Piazza Goidanich 60, 47521 Cesena, Italy; e.urbinati@unibo.it (E.U.); mattia.dinunzio@unibo.it (M.D.N.); gianfranco.picone@unibo.it (G.P.); elena.chiarello2@unibo.it (E.C.); alessandra.bordoni@unibo.it (A.B.); 2 Interdepartmental Centre for Industrial Agri-Food Research (CIRI), University of Bologna, Piazza Goidanich 60, 47521 Cesena, Italy

**Keywords:** in vitro digestion, foodomics, NMR spectroscopy, protein digestibility, carbohydrate digestibility, bioaccessibility, vinegar, balsamic vinegar of Modena, acetic acid

## Abstract

The balsamic vinegar of Modena (BVM), a food specialty under the European Protected Geographical Indication system, is made from grape must blended with wine vinegar exclusively in the Italian province of Modena or Reggio Emilia. Vinegar is associated to an improved digestive function and glycemic response to carbohydrate-rich meals, appetite stimulation, and reduction of hyperlipidemia and obesity. Although many of these effects are attributed to the high concentration of bioactive molecules, the modulation of digestive enzymes activity could have a role. The aim of this study was to investigate the effect of BVM on the digestibility and component release of three foods that are often seasoned with this dressing but have different composition: Parmigiano Reggiano cheese, Bresaola (cured meat), and boiled potatoes. BVM modulated the protein digestion of protein-rich foods (cheese and cured meat) in a matrix-dependent manner, and the BVM effect was mainly related to the inhibition of pepsin in the gastric phase. In the starch-rich food (boiled potatoes), the most impressive effect of BVM was the lower release of anomeric and total carbohydrates, which was consistent with the observed reduction of pancreatic amylase activity. The present investigation shed a new light on the impact of BVM on the digestion process.

## 1. Introduction

Bioaccessibility, that is, the release from the matrix, is a preliminary and important requirement for further absorption of food components. Food component bioaccessibility is not only dependent on their concentration, as food matrix composition and structure also affect the release kinetics during the digestive process [1,2,3,4]. The molecular organization of the food matrix is a determining factor since the spatial distribution of molecules, that is, the supramolecular structure, influences the order of substrate exposure to the digestive enzymes [5]. The food matrix effect is mainly associated to the barrier action exerted by the compartmentalized food structure, which can interfere with the digestion process [6], and it is influenced by processing technologies [7]. Food composition contributes to the matrix effect since the coexistence of different substances in the same food could inhibit or enhance the digestion efficiency, eventually through the modulation of the activity of digestive enzymes [8]. Since any meal includes more than a single food, in addition to the intrinsic food matrix effect, other components coming from other matrices may influence bioaccessibility. The effect of dressing on the accessibility/availability of food components is well known. Examples of this are lemon juice enhancing iron absorption [9,10] and olive oil increasing the bioaccessibility and absorption of lycopene [11,12]. The aim of the present study was to investigate the effect of balsamic vinegar of Modena (BVM), a traditional product from the Emilia Romagna region of Italy, on the bioaccessibility of proteins and carbohydrates embedded in different food matrices. Vinegar is an aqueous solution of acetic acid and trace organic acids, esters, ketones, and aldehydes, which contribute to its organoleptic properties. It is prepared from alcoholic fermentation by yeast, followed by acetous fermentation by acetic acid bacteria, of any suitable food (mainly cereals and fruits). BVM is made from grape must blended with wine vinegar, and it is produced exclusively in the Italian province of Modena and Reggio Emilia, with a Protected Geographical Indication status. In additional to being used as a food ingredient for flavor and functional properties, the potential health benefits of vinegar varieties have led researchers to further consider this food product, used since ancient times [13]. Vinegar has been reported to exert antimicrobial, antioxidant, and antitumor activity, and to regulate blood pressure [13,14]; its effects are mainly ascribed to its content of diverse bioactive compounds including, but not limited to, carotenoids, phytosterols, phenolic compounds, and vitamins C and E. Vinegar is also associated to an improved digestive system function, appetite stimulation, and reduction of hyperglycemia, hyperlipidemia, and obesity [14,15,16,17]. Different mechanisms have been proposed to explain these effects [18], including the modulation of digestive enzymes activity [19,20]. In the present study, the activity of the main digestive enzymes was first measured in the absence and presence of BVM or acetic acid. Then, three different foods were in vitro digested in the absence/presence of BVM. Foods included in the study were Parmigiano Reggiano cheese (PRC), cured meat (Bresaola), and boiled potatoes. BVM is commonly used as dressing in these foods, which have very different compositional characteristics. Potatoes have high carbohydrate content, while Bresaola and PRC are both rich in proteins, which are partially hydrolyzed in the latter product. During in vitro digestion, the extent of the release of soluble molecules from the matrix was assessed by nuclear magnetic resonance (NMR). Protein hydrolysis was also monitored by the Coomassie assay. Although the importance of food matrix and food components on the bioaccessibility of nutrients and metabolites during in vitro digestion has been deeply evaluated [21,22,23,24,25,26,27], to the best of our knowledge, this is the first study aimed to investigate the effect of balsamic vinegar on protein and carbohydrate digestive kinetics and release using an in vitro digestion method.

## 2. Materials and Methods

### 2.1. Chemicals

Unless specified, chemicals and solvents were of analytical grade and purchased from Merck (Darmstadt, Germany) and Sigma-Aldrich (St. Louis, MO, USA).

### 2.2. Pepsin Activity

Pepsin activity was determined according to Anson [28], based on the stop-point assay of hemoglobin degradation. Four hundred microliters of bovine blood hemoglobin (pH 2) was added to 100 µL of acetic acid, BVM, or water solution. The reaction was started with 100 μL of pepsin in a UV transparent cuvette. Final concentration (f.c.) in the assay for hemoglobin, acetic acid, BVM, and pepsin was 1.3% w/v, 0.6% v/v, 10% v/v, and 0.5% v/v, respectively. After 2, 4, 6, 8, or 10 min, the reaction was stopped by adding 1 mL of trichloroacetic acid (TCA), and TCA soluble peptides released from hemoglobin were detected spectrophotometrically at 280 nm (pH 2, 37 °C). Pepsin activity was expressed as Unit, where one unit determines a ΔA280 of 0.001/min, and normalized for milligrams of pepsin in the assay.

### 2.3. Trypsin Activity

Trypsin activity in pancreatin solution was determined according to Hummel et al. [29], based on the stop-point assay of p-toluone-sulfonyl-L-arginine methyl ester (TAME) degradation to p-toluone-sulfonyl-L-arginine for 10 min. One hundred and fifty microliters of TAME (f. c. 1 mM) was added to 1300 μL of reaction buffer (0.046 M Tris, 0.0115 M CaCl_2_, pH 8.1) in a UV transparent cuvette, and the reaction was started with 150 μL of pancreatin solution at three different concentrations (f.c. 0.0125, 0.0250, or 0.05 mg/mL) containing acetic acid (f. c. 0.3% v/v), BVM (f. c. 5% v/v), or water. Trypsin activity was expressed as Unit, where one unit hydrolyses 1 µmole of TAME per minute, and normalized for milligrams of pancreatin in the assay.

### 2.4. Amylase Activity

Amylase activity in 1 mL pancreatin solution (f. c. 15.15 mg/mL) containing acetic acid (f. c. 0.3% v/v), BVM (f. c. 5% v/v), or water was measured using the Amylase Activity Assay kit (Sigma-Aldrich, St. Louis, MO, USA), according to the manufacture’s instruction. Amylase activity was determined using a coupled enzymatic assay, which results in the generation of a chromophoric product (p-nitrophenol, Amax 405 nm) proportional to the amount of substrate (ethylidene-p-nitrophenol-G7) cleaved by the enzyme. Amylase activity was expressed as Unit, where one unit is the amount of amylase that cleaves ethylidene-pNP-G7 to generate 1.0 mmole of p-nitrophenol per minute, and normalized for milligrams of pancreatin in the assay.

### 2.5. Lipase Activity

Lipase activity in 1 mL pancreatin solution (f. c. 15.15 mg/mL) containing acetic acid (f. c. 0.3% v/v), BVM (f. c. 5% v/v), or water was measured using the Lipase Activity Assay kit (Sigma-Aldrich, St. Louis, MO, USA), according to the manufacture’s instruction. Lipase activity was determined using a coupled enzymatic assay, which results in the generation of a product (Amax = 570 nm) proportional to the enzymatic activity in the assay. Lipase activity was expressed as Unit, where one unit is the amount of enzyme that generates 1.0 μmole of glycerol from triglycerides per minute, and normalized for milligrams of pancreatin in the assay.

In all enzymatic assays, the concentration of acetic acid was similar to its concentration in BVM.

### 2.6. In Vitro Digestion

In vitro digestion was performed in triplicate on 20 g of food (PRC, Bresaola, or boiled potatoes) in the presence or absence of 8 mL BVM according to the INFOGEST protocol [30] with slight modifications. To simulate chewing, PRC and Bresaola were coarsely chopped before starting oral digestion. Potatoes of approximately the same size (medium-sized potatoes) were peeled, boiled in water for 20 min, and mashed. In vitro digestion lasted for 245 min (2 min of oral digestion, 120 min of gastric digestion, and 120 min of intestinal digestion) at 37 °C. During the process, several consecutive enzymatic reactions took place by addition of simulated saliva (containing 75 U/mL α-amylase), simulated gastric juice (containing 2000 U/ mL pepsin) at pH 3, and simulated pancreatic juice (containing 10 mM bile and 100 U/mL pancreatin) at pH 7. Final volume of oral, gastric, and duodenal digestion was 20, 40, and 80 mL, respectively. Samples (10 mL each) were taken every hour during the gastric phase (1G and 2G), and every 30 min during the duodenal phase (1D, 2D, 3D, and 4D). All samples were stored at −80 °C until further analysis.

### 2.7. HR-NMR Spectroscopy

Sample preparation: Samples were thawed, centrifuged first at 2300 × *g* for 5 min at 4 °C to eliminate the coarser particles and then at 50,000× *g* for 5 min at 4 °C to eliminate the fine particulates. Afterward, each sample was vortexed for 30 **s**, then three 1 mL aliquots were taken. Aliquots were centrifuged at 18,600× *g* for 10 min at 4 °C, then 900 μL of supernatant was taken and added to 160 μL of 100 mM phosphate buffer with 10 mM trimethylsilylpropanoic acid (TSP), molecular weight (MW) 172.27 g/mol (Cambridge Isotope Lab Inc., Tewksbury, MA, USA) and brought to pH 7.0. Before analysis, samples were centrifuged again at 18,600× *g* for 10 min at 4 °C to remove possible particles, and 900 μL of supernatant was used for analysis. HR-NMR spectrum acquisition parameters: Spectra were acquired according to Picone et al. [31]. HR-NMR spectra were recorded at 300 K on a Bruker US+ Avance III spectrometer operating at 600 MHz, equipped with a BBI-z probe and a SampleCase™ sampler for automation. The spectra were collected with a 90° pulse of 13 µs with 7.7 W of power, a relaxation delay of 4 s, and an acquisition time of 2.5 s. For each sample, 32 scans were collected into 32 K data points covering a 20 parts-per-million ppm spectral width. The mono-deuterated water (HOD) residual signal was suppressed by applying the NOESYGPPR1D sequence (a standard pulse sequence included in the Bruker library) incorporating the first increment of the NOESY pulse sequence and a spoil gradient [32]. The data were Fourier-transformed into 64 K data points. Phase and baseline corrections were automatically performed using TopSpin version 3.0 (Bruker BioSpin, Karlsruhe, Germany). The chemical shifts were internally referenced to the TSP at 0.000 ppm. The spectra were normalized to the TSP area in order to correct vertical scale errors due to the incremental concentration of solubilized molecules upon digestion. The HR-NMR spectra acquired on the samples of digestion fluid, at the different digestion steps, were partitioned into 5 different regions, collecting signals from different classes of compounds, according to the same approach used in previous digestion studies conducted on Bresaola and PRC [33,34]. In detail, region A (0.40–1.10 ppm) was selected as collecting the aliphatic signals belonging to the methyl groups, in particular those of branched amino acids; region B (3.00–4.50 ppm) included the signals originating from carbohydrates; region C (4.16–4.56 ppm) included the alpha-hydrogen signals of amino acids; region D (6.80–7.50 ppm) and region E (7.30–9.00 ppm) included signals of aromatic side chains of amino acids and of hydrogen atoms of the peptide bond not accessible to water, respectively. It is worth noting that the total area of region C was selected as being proportional to the total amount of soluble amino acids (free and bound to peptides and proteins). Signals belonging to the enzymes added to the digestion system were subtracted. NMR spectra were acquired in triplicate on three independent digestion repetitions.

### 2.8. Protein Quantitative Analysis

Protein content was determined by the Coomassie assay using bovine serum albumin as standard, as previously described [35].

### 2.9. Statistical Analysis

Statistical analysis was applied by the one-way analysis of variance (ANOVA) followed by Tukey’s post hoc test to compare enzymes activity in the presence/absence of acetic acid and BVM, and the release of soluble molecules at different time points of digestion. The effect of BVM at each time point of digestion and the difference of integral area between the first (1G) and last (4D) time point of in vitro digestion were evaluated by the Student’s *t*-test considering *p* < 0.05 as significant.

## 3. Results and Discussion

The activity of digestive enzymes in the absence/presence of acetic acid or BVM was evaluated at a concentration proportional to their concentration in digestive fluids during in vitro digestion.

As reported in Table 1, BVM significantly reduced pepsin and pancreatic amylase activity and increased lipase activity, while acetic acid decreased amylase activity without any effect on other enzymes.

We speculate that the reduction of pancreatic amylase activity was mainly due to pH lowering, as recently observed by Freitas et al. [20] on salivary amylase and by Ahmadniaye Motlagh et al. on pancreatic amylase [19]. Indeed, polyphenols could contribute to the effect. Comparing different vinegars from grains and fruits, Noh et al. observed a decrease in α-amylase activity, the inhibitory effect being higher in vinegars with high organic acid and phenolic content [36]. Although an inhibitory effect of some polyphenols has been reported also on pepsin activity [37,38,39,40], and balsamic vinegar possesses the highest concentration of polyphenols among fruit vinegars [41], we did not observe any modification in trypsin activity. Monomers of tannins such as catechins and gallic acid have been shown to be 1000 times less active in the inhibition of trypsin compared with high-molecular-weight tannic acid [42]. Gallic acid is the most concentrated polyphenol in BVM [43], and this could explain, at least in part, the lack of trypsin inhibition.

The observed modulation of pancreatic lipase by BVM is consistent with the recent study by Ahmadniaye Motlagh et al. [19], who reported that apple cider vinegar administration to fish slightly increases lipase activity.

Evaluation of the activity in an enzymatic assay is not sufficient to predict the effects of the addition of BVM during digestion of real foods. Food components behave differently in isolated form than when forming the food structures, and the “matrix effect” is an aspect to be carefully considered for defining properties and derived nutritional/health implications [7]. To verify whether the food matrix modulates the effect of BVM, two protein-rich foods (PRC and Bresaola) and one starch-rich food (boiled potatoes) were in vitro digested. The progression of in vitro digestion of the three food matrices was evaluated in the absence/presence of BVM considering the appearance of new signals in six NMR spectral regions:

(i) The aromatic region, where the signals of tyrosine, phenylalanine, and tryptophan resonate;
(ii) The aliphatic region, where the signals of all the branched chain amino acids (BCAAs) fall;
(iii) The α-amino acid proton region, which includes the signals of all the amino acids (AAs), including those mentioned above, both in the free state and bound to peptides or in soluble proteins;
(iv) The peptide proton region, collecting the amide signals that are not accessible to water. They are part of structured proteins or macropeptides that hide the peptide bonds inside the globular structure. Therefore, small peptides, which necessarily expose this group, and single AAs, which do not have the peptide bond, are not providing signals in this region.

**Figure 1:**
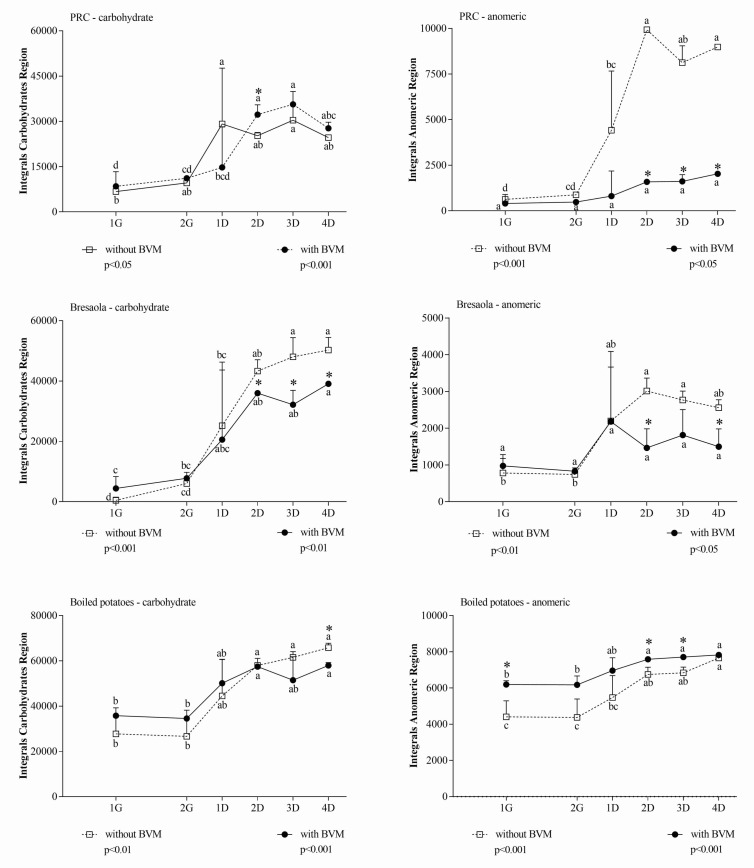
Integral area of carbohydrate and anomeric region of in vitro digested samples of Parmigiano Reggiano cheese (PRC), Bresaola, and boiled potatoes. All data are means ± SD of at least three independent in vitro digestions. In each food matrix and spectral region, statistical analysis was by the one-way ANOVA with Tukey’s post hoc test to compare the release of soluble molecules at different time points (different letters indicate significant differences) and by the Student’s *t*-test to evaluate the effect of BVM at each time point (* at least *p* < 0.05).

The extent of protein hydrolysis was also followed using the Coomassie assay; we adopted an analytical approach based on NMR spectroscopy coupled to a colorimetric assay since they provide complementary information about the size of protein fragments released during the different phases of digestion. In this context, we refer to metabolites as molecules originating from digestion reactions. In addition, to clarify the overall impact of BVM on the digestion process, in each spectral region the difference of integral area between 1G and 4D was also calculated. The latter differential measurement excludes possible interfering signals originating from the BVM that are not related to the release of soluble species from the food matrix during the digestion. In each spectral region, the effect of BVM was dependent on the considered food matrix. In the carbohydrate and anomeric region (Figure 1), considering the higher sugar concentration when samples were digested in the presence of BVM, ∆ values between G1 and other time points in the same condition were more representative than the comparison at the same time point in the presence/absence of BVM. Differential values indicated that BVM had almost no significant effect during gastric digestion of any food, while it decreased metabolite release during duodenal digestion.

The overall inhibition of metabolite release in the carbohydrate and anomeric region due to BVM addition during in vitro digestion was confirmed measuring differential integral areas between 1G and 4D (Table 2).

**Figure 2:**
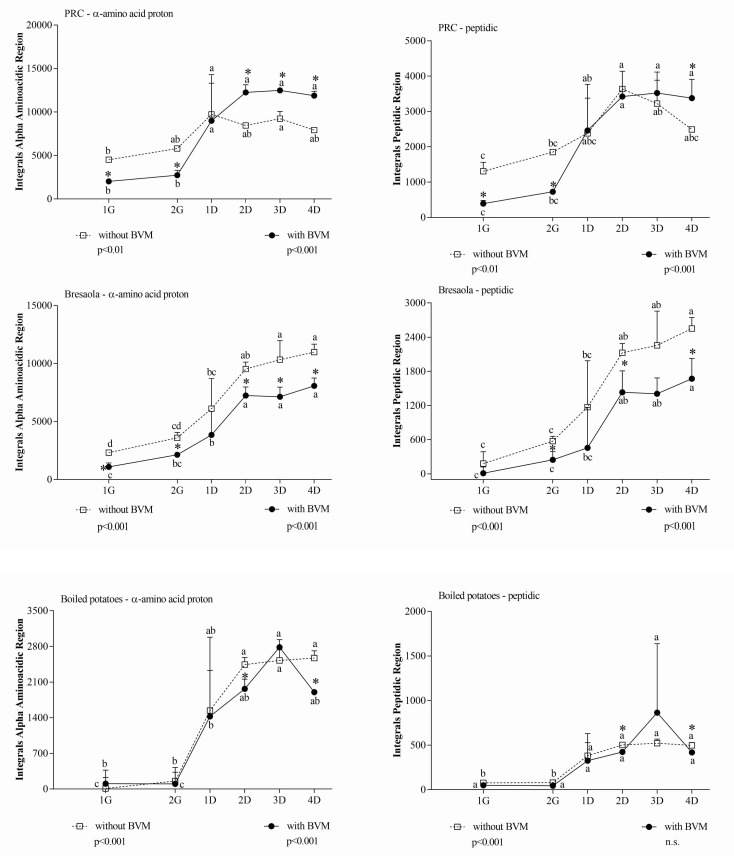
Integral area of α-amino acid and peptide proton regions of in vitro digested samples of PRC, Bresaola, and boiled potatoes. All data are means ± SD of at least three independent in vitro digestions. In each food matrix and spectral region, statistical analysis was by the one-way ANOVA with Tukey’s post hoc test to compare the release of soluble molecules at different time points (different letters indicate significant differences) and by the Student’s *t*-test to evaluate the effect of BVM at each time point (* at least *p* < 0.05).

In the α-amino acid and peptide proton regions, differences detected between samples at the same time point were not ascribable to substances already present in BVM, since the signals generated by BVM alone in those regions were negligible. In PRC, during the gastric phase, BVM significantly inhibited the release of metabolites, while its effect was opposite during the duodenal phase. In Bresaola and boiled potatoes, BVM addition had almost no effect during the gastric digestion, while it reduced the metabolites release during the duodenal phase (Figure 2).

**Figure 3:**
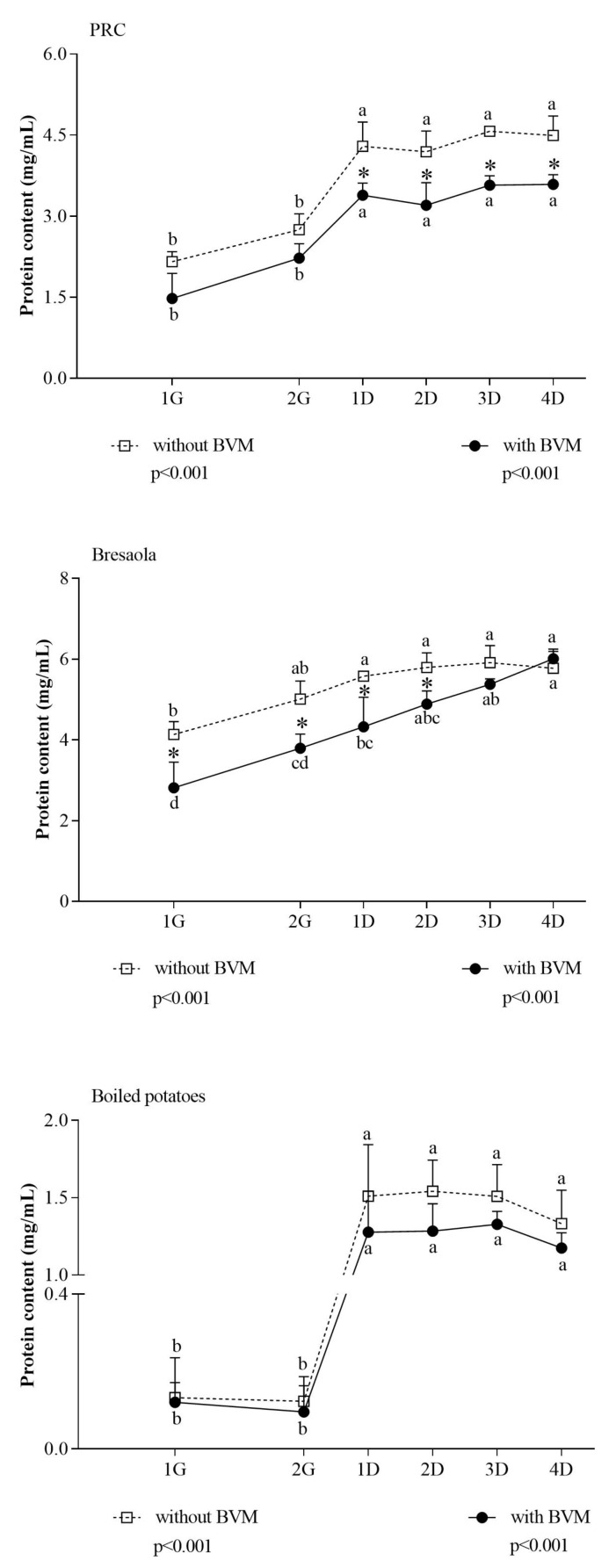
Extent of protein hydrolysis in PRC, Bresaola, and boiled potatoes at different time points of in vitro digestion. Protein hydrolysis into peptides >3 KDa was evaluated by the Coomassie assay. Data are means ± SD of at least three independent in vitro digestions. In each food matrix, statistical analysis was by the one-way ANOVA with Tukey’s post hoc test to compare protein hydrolysis at different time points (different letters indicate significant differences with a *p* < 0.05), and by the Student’s *t*-test to evaluate the effect of BVM at each time point (* at least *p* < 0.05).

In Bresaola and boiled potatoes, results obtained with the Coomassie assay were consistent with NMR results (Figure 3). In PRC, the apparent discrepancy between the two methods could simply reflect the inability of the colorimetric method to detect small peptides [44]. Comparing results from the two methods, we speculated that a large number of proteins were further hydrolyzed to <3 KDa peptides, which are detected by NMR only, during the duodenal phase.

The comparison among the differential spectral areas (∆ 4D–1G) in the three matrices (Table 3) evidenced that BVM had an overall permissive effect on the release of small peptides only in PRC. In this matrix, the wide differential for both α-amino acid proton region and peptide region can be attributed to a combined effect of inhibition of protein hydrolysis exerted by BVM during gastric digestion, followed by an enhancement of protein hydrolysis, consisting mainly of the release of small peptides not detected by the Coomassie assay, during the duodenal phase. The similar release of large peptides during the duodenal phase in the absence/presence of BVM, as inferred from the inspection of the NMR peptide region where the signals come only from large peptides and protein fragments, confirms this hypothesis.

**Figure 4:**
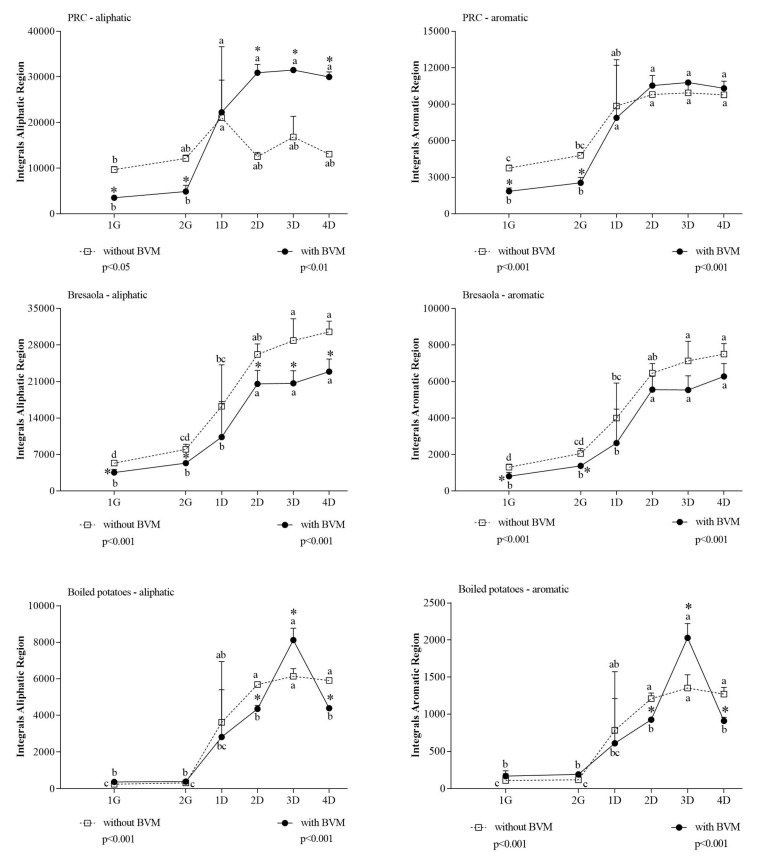
Integral area of aliphatic and aromatic region of in vitro digested samples of PRC, Bresaola, and boiled potatoes. All data are means ± SD of at least three independent in vitro digestions. In each food matrix and spectral region, statistical analysis was by the one-way ANOVA with Tukey’s post hoc test to compare the release of soluble molecules at different time points (different letters indicate significant differences) and by the Student’s *t*-test to evaluate the effect of BVM at each time point (* at least *p* < 0.05).

Exploiting the specificity of some diagnostic spectral regions, NMR spectroscopy also allowed the evaluation of the average amino acid composition of the peptides released from the matrix and solubilized in the digestion fluid. Comparing integral areas in the aliphatic and aromatic regions (Figure 4) with those associated to the general α-amino acid protons, it was possible to estimate the composition of the released peptides.

The comparison among the differential spectral areas between 1G and 4D (Table 4) in the three matrices evidenced that BVM had a permissive effect on the release of aliphatic and aromatic amino acids only in PRC.

In PRC, BVM addition caused a different progression in the increase of aromatic and aliphatic signals compared with control digestion. In the aliphatic region, which is associated to BCAAs, BVM had an inhibitory effect during the gastric phase while it caused a steep enhancement in the duodenal phase. The inspection of the aromatic region evidenced during the duodenal phase a bigger release of the corresponding amino acids in the presence of BVM. This increase was higher than expected considering the trend of the α-amino acid proton region (Figure 2). While for PRC we evidenced a different amino acids composition for peptides released in the presence/absence of BVM, this differential effect was not evidenced in Bresaola and boiled potatoes, being the two curves relative to aromatic and aliphatic protons areas similar to those observed for the generic α-amino acids protons. The different impact of BVM addition could be related to the different composition and structure of proteins in cheese (mainly caseins), Bresaola (myofibrillar proteins), and potatoes (plant proteins, mainly patatin).

Overall, results obtained in the present study clearly indicated that protein digestion of protein-rich foods is modulated by BVM in a matrix-dependent manner. The effect of BVM on protein digestibility seemed mainly related to the inhibition of pepsin in the gastric phase. Notably, pepsin activity was not inhibited by acetic acid. BVM inhibition of pepsin activity had a lower impact on gastric digestion of PRC, whose proteins are already partially hydrolyzed due to lactic fermentation [45]. Although BVM inhibited gastric hydrolysis of proteins in Bresaola, which is characterized by larger muscular protein in compartmentalized fibers, it caused no inhibition of further protein hydrolysis to small peptides during the duodenal phase. The overall bioaccessibility of Bresaola proteins was increased, allowing us to conclude that pepsin inhibition by BVM had no effect on protein digestibility although generating a different digestion pathway. In boiled potatoes, although the hydrolysis in larger protein fragments was inhibited by BVM, the overall protein digestibility (i.e., total amount and composition of released peptides) was not affected. In this starch-rich food, the most impressive effect of BVM was related to the much lower release of anomeric and total carbohydrates. This is consistent with the observed reduction of pancreatic amylase activity in the presence of acetic acid and BVM, and further confirms that the inhibition of the enzyme is one of the possible pathways by which vinegar may improve the glycemic response to carbohydrate-rich meals [46].

The composition of BVM was previously reported [47], and we did not characterize it in the present study. Although we cannot exclude that differences in the composition, even if small due to strict disciplinary of production, could modulate the effect of BVM on digestion, it is conceivable that their impact on the overall effect is small compared with the effect of the food matrix.

## 4. Conclusions

The influence of the food matrix on the bioaccessibility of food components has been widely reported. The significance and complexity of components’ interactions within the food matrix [48] as well as the food structure and texture [49] and the processing [50] have been considered as variables influencing bioaccessibility. The approach applied in the present investigation shed a new light on the relation between the composition of the food and the digestion process, including also the seasoning as a variable. On the one side, this approach was useful to better exploit the nutritional properties of some products such as BVM that are often considered only accessory components added to the recipe for their sensorial properties. In a broader, foodomics vision of the interactions of the food and nutrition domains, it represents a step ahead to build up models considering as a whole the different foods composing the meal, so reproducing more accurately what happens in vivo and emphasizing that, for the purposes of optimal nutrition, it is necessary to consider the whole diet rather than the single food.

## Figures and Tables

**Table 1:** Digestive enzymes activity in the absence/presence of acetic acid and balsamic vinegar of Modena (BVM). Data are means ± SD of six replicates. Statistical analysis was by the one-way ANOVA (pepsin: *p* < 0.05; amylase and lipase: *p* < 0.001) with Tukey’s post hoc test. Different letters in the same row indicate significant difference (at least *p* < 0.05).

	Pepsin	Pepsin with acetic acid ( 0.6% v/v)	Pepsin with BVM ( 10% v/v)
Pepsin activity (U/mg)	1926.61 ± 642.70 ^a^	1945.22 ± 615.57 ^a^	958.5 ± 526.13 ^b^
	**Pancreatin**	**Pancreatin and acetic acid (f.c. 0.3% v/v)**	**Pancreatin and BVM (f.c. 5% v/v)**
Trypsin activity (U/mg)	16.36 ± 2.62 ^a^	16.72 ± 2.16 ^a^	15.88 ± 1.73 ^a^
Amylase activity (U/mg)	27.25 ± 1.55 ^a^	3.14 ± 0.45 ^c^	8.12 ± 1.9 ^b^
Lipase activity (mU/mg)	1.37 ± 0.09 ^b^	1.34 ± 0.39 ^b^	5.96 ± 0.6 ^a^

**Table 2:** Differential integral area (∆ 4D–1G) of carbohydrate and anomeric region of in vitro digested PRC, Bresaola, and boiled potatoes. All data are means ± SD of at least three independent in vitro digestions. Statistical analysis was by Student’s *t*-test.

Region	Without BVM	With BVM	*p*
**PRC**			
Carbohydrate	18,928 ± 3008	22,754 ± 194	0.092
Anomeric	8357 ± 746	2439 ± 407	0.0003
**Bresaola**			
Carbohydrate	50,725 ± 3755	39,106 ± 4310	0.024
Anomeric	2374 ± 244	1210 ± 575	0.032
**Boiled potatoes**			
Carbohydrate	40,369 ± 5376	22,398 ± 2508	0.006
Anomeric	3416 ± 707	1760 ± 323	0.021

**Table 3:** Differential integral area (∆ 4D–1G) of α-amino acid and peptide proton regions of in vitro digested PRC, Bresaola, and boiled potatoes. All data are means ± SD of at least three independent in vitro digestions. Statistical analysis was by Student’s *t*-test.

Region	Without BVM	With BVM	*p*
**PRC**			
α-Amino acid proton	3443 ± 242	10,433 ± 935	0.0002
Peptide	851 ± 136	3212 ± 457	0.001
**Bresaola**			
α-Amino acid proton	7295 ± 843	6368 ± 340	0.153
Peptide	2220 ± 123	1751 ± 307	0.070
**Boiled potatoes**			
α-Amino acid proton	2546 ± 33.76	1975 ± 432	0.080
Peptidic	429 ± 16	488 ± 108	0.625

**Table 4:** Differential integral area (∆ 4D–1G) of aliphatic and aromatic region of in vitro digested PRC, Bresaola, and boiled potatoes. All data are means ± SD of at least three independent in vitro digestions. Statistical analysis was by Student’s *t*-test.

Region	Without BVM	With BVM	*p*
**PRC**			
Aliphatic	3758 ± 1084	28,001 ± 2546	0.0001
Aromatic	6107 ± 386	9018 ± 771	0.004
**Bresaola**			
Aliphatic	25,644 ± 1536	20,311 ± 2556	0.036
Aromatic	6368 ± 527	5718 ± 718	0.275
**Boiled potatoes**			
Aliphatic	5975 ± 693	5110 ± 2028	0.523
Aromatic	1264 ± 281	1049 ± 602	0.605

## Data Availability

Datasets generated and analyzed during the study, supporting the reported results, are available, upon request via email to F.C., by the Bio-NMR lab.
